# The Emerging Role of Macrophages in Chronic Cholangiopathies Featuring Biliary Fibrosis: An Attractive Therapeutic Target for Orphan Diseases

**DOI:** 10.3389/fmed.2020.00115

**Published:** 2020-04-21

**Authors:** Massimiliano Cadamuro, Noemi Girardi, Gregory J. Gores, Mario Strazzabosco, Luca Fabris

**Affiliations:** ^1^Department of Molecular Medicine, University of Padua, Padua, Italy; ^2^Division of Gastroenterology and Hepatology and the Mayo Clinic Center for Cell Signaling in Gastroenterology, Mayo Clinic, Rochester, NY, United States; ^3^Liver Center, Department of Medicine, Yale University, New Haven, CT, United States

**Keywords:** primary sclerosing cholangitis, congenital hepatic fibrosis, monocyte, cholangiocyte, biliary repair

## Abstract

Cholangiopathies are a heterogeneous group of chronic liver diseases caused by different types of injury targeting the biliary epithelium, such as genetic defects and immune-mediated attacks. Notably, most cholangiopathies are orphan, thereby representing one of the major gaps in knowledge of the modern hepatology. A typical hallmark of disease progression in cholangiopathies is portal scarring, and thus development of effective therapeutic approaches would aim to hinder cellular and molecular mechanisms underpinning biliary fibrogenesis. Recent lines of evidence indicate that macrophages, rather than more conventional cell effectors of liver fibrosis such as hepatic stellate cells and portal fibroblasts, are actively involved in the earliest stages of biliary fibrogenesis by exchanging a multitude of cues with cholangiocytes, which promote their recruitment from the circulating compartment owing to a senescent or an immature epithelial phenotype. Two cholangiopathies, namely primary sclerosing cholangitis and congenital hepatic fibrosis, are paradigmatic of this mechanism. This review summarizes current understandings of the cytokine and extracellular vesicles-mediated communications between cholangiocytes and macrophages typically occurring in the two cholangiopathies to unveil potential novel targets for the treatment of biliary fibrosis.

## Introduction

Cholangiopathies are a heterogeneous group of liver diseases targeting the biliary epithelium of different etiologies including genetic defects (causing Alagille syndrome, cystic fibrosis-related cholangiopathy, and polycystic and fibropolycystic liver diseases) and immune-mediated attacks (causing primary biliary cholangitis and primary sclerosing cholangitis) (1–3). Their clinical evolution is generally much slower than liver diseases aimed at the hepatocytes, spanning over years or decades, and depends on the balance between the manifestations related to the bile duct injury such as cholestasis, ductopenia (or conversely, exuberant bile duct expansion), and portal/biliary fibrosis (4). Whereas, cholestasis related to the impaired ability of the biliary epithelium to alkalinize or fluidify the primary bile produced by the hepatocytes is a common manifestation observed in most of them, biliary fibrosis is a variable occurrence (5). However, if present, biliary fibrosis is a major determinant of disease progression as it is responsible for the disease's most feared complications such as portal hypertension and biliary cirrhosis, and it bears an increased risk of malignant transformation into cholangiocarcinoma (6, 7). Clinically, management of cholangiopathies still represents one of the major gaps in modern hepatology knowledge in terms of both early detection and effective treatment (3). To date, only a few pharmacological therapies have been proven to be effective and only in limited pathology conditions (urosodeoxycholic acid, obeticholic acid, and bezafibrate in primary biliary cholangitis), while the vast majority of cholangiopathies are still orphan. Liver transplantation remains the only option when the disease is advanced (8–10). Major efforts for developing effective therapeutic strategies in cholangiopathies should be devoted to halt biliary fibrogenesis (4, 11). For many years, understanding of the mechanisms of biliary fibrosis has relied on studies performed in animal models where biliary fibrosis develops in a rapid manner not consistent with what is observed in humans (such as the bile duct ligation in rodents), or in animal models where the primary dysfunction affects the hepatocyte rather than the cholangiocyte [such as the *multidrug resistance* (*mdr)2*-KO mouse] (12–14). These studies have lent support to the notion that biliary fibrogenesis is an intricate process where multiple cell types are actively involved. Following biliary injury, cholangiocytes lining the finest ramifications of the biliary tree, abutting the canals of Hering, become activated in a so-called “ductular reaction” (2, 15–17). They start to secrete a huge variety of mediators like cytokines, chemokines, and growth factors, and to express a rich repertoire of receptors that enable them to communicate extensively with portal fibroblasts and hepatic stellate cells, the ultimate effectors of the deposition of new extracellular matrix (ECM) components (2, 18). Cell interactions between the epithelial and the mesenchymal compartments are molded by the further involvement of inflammatory cell types recruited from the blood such as neutrophils, macrophages, and lymphocytes (19, 20). Among them, macrophages behave as emerging players in biliary fibrogenesis since the initial phase, and deciphering their involvement has provided insights with translational significance (19, 21, 22). Therefore, the aim of the present review is to draw attention to some recent observations highlighting the role played by macrophages in biliary fibrosis, derived from experimental models of bile duct injury with progressive portal scarring, which recapitulates the phenotype of primary sclerosing cholangitis (PSC) and congenital hepatic fibrosis (CHF), as they may shed light on novel therapeutic approaches.

## General Concepts on the Involvement of Macrophages in Fibrosis

Macrophages are a cellular population extensively represented in the liver that display a wide heterogeneity dependent upon their developmental origin (resident or infiltrating macrophages) and their polarization (inflammatory or anti-inflammatory) regulated by microenvironmental cues, such as danger signals, and cellular debris taken up by phagocytosis. In normal conditions, liver-resident macrophages, or Kupffer cells (KC), are mainly localized in the perisinusoidal space to ensure tissue homeostasis, antimicrobial defense, and proper metabolism (23, 24). In disease conditions, macrophages play a central role in host defense against infections as well as in tissue repair (25). In these cases, infiltrating macrophages can be recruited following mobilization of circulating monocytes from bone marrow and the spleen. In mouse, the tissue origin of infiltrating macrophages can be assessed by a distinct phenotype. In fact, bone marrow-derived monocytes are Ly-6C high (Ly-6C^hi^) and express several receptors such as C-C chemokine receptor type 2 (CCR2) and receptors for pathogen and damage associated molecular patterns (PAMPs and DAMPs, respectively), while the spleen-derived macrophages are Ly-6C low (Ly-6C^lo^) and are less equipped with the receptor machinery (26). Moreover, macrophages possess a high level of cell plasticity, enabling them to respond to a huge variety of both endogenous and exogenous stimuli, interact with multiple cell types, and direct mechanisms underlying tissue repair and regeneration. Following ligand interaction with its receptor(s), macrophages activate a number of cellular responses including phagocytosis, endocytosis, and secretion of soluble mediators like cytokines, chemokines, and growth factors (27, 28). Thanks to these properties, once stimulated, macrophages may exert a range of functions including cell adhesion and migration, antigen presentation, and effector functions in the immune response. Exogenous ligands are bound by receptors involved in the recognition of opsonins, mainly antibodies produced by the complement activation, or through the direct recognition of carbohydrates, proteins, lipids and nucleic acids. In particular, stimulation of different Toll-like receptors (TLR) and lectins expressed by macrophages converge toward the transcription factor NF-kB, whose activation regulates production of proinflammatory and profibrotic mediators (29). Macrophages can also express the receptor for advanced glycation end products (RAGE), a multi-ligand receptor that can bind, among others, the alarmin high-mobility group box-1 (HMGB1), belonging to the DAMP group, and S100 (30). Similar to TLR, RAGE interaction with HMGB1 kindles the pro-inflammatory cascade regulated by NF-kB, with a partial overlap between RAGE and TLRs, since RAGE-deficient macrophages showed a decrease cytokine response following HMGB1 stimulation (29). In response to signals released in an “inflamed” environment, macrophage activation displays marked functional changes that can be roughly divided into M1 (classically activated) or M2 (alternatively activated) phenotypes as the edges of a continuum without a clear demarcation, dependent on the phase and the degree of tissue injury (31). Macrophages undergo M1 activation in the presence of Th1 lymphocytes or upon the effect of multiple pro-inflammatory cytokines, thus increasing their ability to kill intracellular pathogens and contributing to the progression of the inflammatory process. In particular, M1 macrophage differentiation is induced in response to interferon-γ, exposure to microorganisms or microbial products such as lipopolysaccharides (LPS), and cytokines such as tumor necrosis factor-α (TNF-α) (32). M1 macrophages secrete low levels of interleukin (IL)-10 and high levels of IL-1β, IL-6, IL-12, IL-23, and the TNF-α itself, resulting in further activation of Th1 effector cells, thus sustaining a feed-forward loop (33, 34). Beside the secretory profile, the M1 polarization can be identified also by a variety of functional and biochemical phenotypic markers including the overexpression of surface markers, in particular MHC class II and CD86, and the enhanced competence for antigen presentation and microbicide activity (35). Pathogen elimination is indeed facilitated by an increased production of reactive oxygen species (ROS) and nitric oxide (NO), due to the activation of the inducible nitric oxide synthase (iNOS) (36, 37). On the other side of the spectrum, exposure to anti-inflammatory cytokines induces macrophages toward an alternatively activated or M2 phenotype, with increased expression of Ly-6C (Ly-6C^hi^) and of specific markers such as CD206, Arginase-1, and early growth response gene-2 (Egr2) (38, 39). M2 macrophages do not possess antimicrobial activity due to their inability to produce NO and ROS, and despite the fact that they may express MHC class II proteins, they are inefficient as antigen-presenting cells. Rather, they mostly inhibit the proliferation of T cells, and cooperate with the humoral immune response by regulating the Th2 effectors and stimulating the secretion of immunoglobulins by B cells. Classical inducers of M2 polarization are IL-4 or IL-13, featuring the Th2 response (32, 37). In particular, IL-4 activates the Janus kinase/signal transducer and activator of transcription protein 6 (JAK/STAT6) pathway, which leads to the transcription of several genes, among them *Arachidonate 15-Lipoxygenase, C-C Motif Chemokine Ligand (CCL)22, CCL26, CD23a, Fibronectin 1*, and *suppressor of cytokine signal*, regulating inflammatory cell recruitment and activation and ECM deposition (40, 41). Furthermore, IL-4-stimulated macrophages showed an increased production of anti-inflammatory cytokines (IL-10 and IL-1R antagonist) in conjunction with a reduced expression of proinflammatory/M1-like cytokines (IL-1, TNFα, IL-6, IL-12, and MIP1α), an essential step to dampen inflammation. Furthermore, in animal models of both acute (42, 43) and chronic liver diseases (44), the ability of M2 macrophages to blunt inflammation also relies on the stimulation of apoptosis in M1 macrophages via a IL-10-mediated mechanism, which affects the balance between the anti- and pro-apoptotic proteins Bax and Bcl-2. In addition to the immunomodulatory functions, M2 macrophages are pivotal players in tissue regeneration and repair given their contribution to angiogenesis and ECM remodeling (45). M2 macrophages may stimulate angiogenesis as they secrete high levels of vascular endothelial growth factor (VEGF)-A (45). Furthermore, M2 macrophages can also produce major components of the “scarring” ECM, including fibronectin and collagen, as shown by up-regulation of *Col1a2* and *Col3a1* genes (46). On the other hand, the constitutive activation of arginase in M2 macrophages leads to *de novo* biosynthesis of polyamine and proline, which promotes cell growth and further deposition of collagen in an autocrine loop (47). Moreover, M2 macrophages are responsible for the degradation of the native constituents of ECM by secreting matrix metalloproteinase −9, −12, and −13. While the role of macrophages in liver fibrosis developing in the setting of a parenchymal damage has been extensively studied and has been the subject of recent reviews (21), evidence on their significant contribution to biliary fibrosis have only recently been emerging. Accumulation of macrophages is a distinctive trait of biliary fibrosis, in particular when it is particularly prominent (PSC, CHF). Of note, these conditions share some relevant clinical aspects, as both disorders may develop portal hypertension without progression to full-blown biliary cirrhosis and bear an increased risk of malignant progression toward cholangiocarcinoma.

## The Fibrogenic Role of Macrophages in PSC

Among chronic liver diseases, PSC is paradigmatic of the pathophysiological relevance of progressive fibrogenesis, which generates a tight and stiff sheath around the ductal epithelium whereby necroinflammation is less pronounced compared with other inflammatory cholangiopathies. The hallmark of the disease is in fact the development of concentric periductal fibrosis, leading to the narrowing and eventual obliteration of both small and large bile ducts, which at the radiological level results in the formation of diffuse multifocal biliary strictures recognizable as a “beaded” configuration (48). The pathogenesis of PSC is largely unknown but immune-mediated mechanisms are likely involved, although the nature of the triggering factors remains elusive. The frequent association with inflammatory bowel disease, mainly chronic ulcerative colitis, suggests that bacterial components enriched in endotoxins like LPS and delivered to the liver parenchyma through the portal circulation may also be relevant in the pathogenesis of PSC (49). Interesting insights into the mechanisms responsible for biliary fibrosis can derive from a deep phenotyping of the portal cell infiltrate. Using a high-throughput sequencing approach, Govaere et al. found that in PSC, the peribiliary milieu was extensively populated by CCL28^+^ macrophages already in the early stage of the disease before developing advanced fibrosis in contrast to HCV chronic hepatitis, which instead showed an enrichment in T and B cells in the areas of hepatocellular injury and regeneration (50). Macrophage recruitment to the biliary microenvironment has been confirmed in murine models of PSC (*Mdr2*^−/−^ mouse), where they promote injury and cholestasis (51). Of note, the immunophenotype of macrophages accumulating in the periportal areas of liver sections obtained from PSC patients and *Mdr2*^−/−^ mice, is consistent with an origin from circulating monocytes rather than from the resident KCs (CD68^+^/CCR2^+^ in human, CD45^+^/F4/80^+^/CD11b^hi^ in mice). In PSC, following the initial pro-inflammatory reaction, the macrophage infiltration of the peribiliary area increased as the fibrotic stage of the disease progressed, encompassing both pro-inflammatory iNOS^+^ M1 and anti-inflammatory CD206^+^ M2 phenotypes, with a predominance of M1 in advanced stages (51).

### Cell Senescence and Extracellular Vesicle Release Are Distinctive Epithelial Traits Promoting Macrophage Recruitment

**Figure 1:**
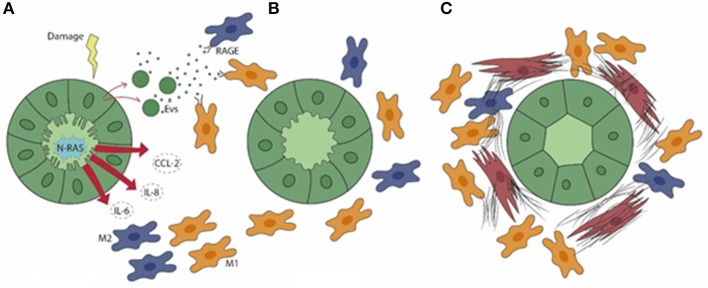
In PSC **(A)**, senescent cholangiocytes harbor activation of N-Ras signaling, resulting in the secretion of the pro-inflammatory mediators IL-6, IL-8, and CCL2, which drive the peribiliary accumulation of both M1 (yellow) and M2 (blue) macrophages. Extracellular vesicles (EVs) containing damage-associated molecular patterns (DAMPs) are also released by senescent cholangiocytes. **(B)** By binding the RAGE receptor expressed by macrophages, EVs further elicit macrophage activation and infiltration. **(C)** M1 macrophages become preponderant in a later phase, when the generation of a dense fibrotic tissue is further enhanced by the accumulation of portal myofibroblasts (brown).

In PSC, a distinctive feature of cholangiocytes is cell senescence, an irreversible condition of cell cycle arrest. Senescence is induced by the activation of the N-Ras pathway upon persistence of cellular injury (52), where alterations in cell morphology, increased lysosomal activities, and enhanced DNA damage responses are associated with exuberant pro-inflammatory secretory functions, which involve IL-6, IL-8, CCL2, and plasminogen activator inhibitor 1, and collectively result in the senescence-associated secretory phenotype (SASP). Interestingly, SASP activation in PSC is closely associated with macrophage recruitment. *In vitro* models of cellular senescence, generated by treating normal human cholangiocytes with the inhibitor of apoptosis antagonist BV6 or with LPS, and cholangiocytes isolated from PSC livers, released monocyte chemotactic factors including CCL2/monocyte chemoattractant protein (MCP)-1 and IL-8, regulated by the transcription factor NF-kB, that promoted monocyte migration (51). In particular, strategies aimed at blocking CCR2, the cognate receptor of CCL2/MCP-1, by pharmacological treatment with cenicriviroc (dual antagonist of CCR2/5) or by genetic ablation led to a significant therapeutic gain in mouse models of PSC *in vivo*. In these conditions, the reduction in peribiliary infiltration of monocyte-derived macrophages was paralleled by a stark improvement of fibrosis as well as of cholestatic indexes (53). These findings were reproduced in an experimental model of acute sclerosing cholangitis generated by biliary instillation of BV6, where targeting CCR2-dependent monocyte recruitment significantly attenuated both the inflammatory and fibrotic responses, thus highlighting the concept that macrophage accumulation within the periductal space is a key event since the initial stages of biliary injury (51). Recent data showed that in addition to soluble factors, cholangiocytes can activate macrophages originating from the bone marrow by releasing membrane-derived nanometer-sized (from 40 to 200) extracellular vesicles (EVs) (54). EVs are functionally active, bilayer-delimited particles including miRNA and DAMPs which bear several small biomolecules mediating cell-to-cell communication as a sort of “secret messengers” (55). Apoptotic bodies from dying cells are usually larger than 500 nm, thus EV-mediated DAMP cargo is of particular interest in conditions with negligible cell death by necrosis, as occurred in PSC. Among DAMP, S100A11 is contained in EVs released by primary cholangiocytes derived from *Mdr2*^−/−^ mouse and grown in 3D-structures as cholangioids (54). Of note, S100A1 is a potent agonist of RAGE (56) and highly expressed by macrophages. Upon binding to S100A1, bone marrow-derived macrophages turned to a proinflammatory phenotype involving secretion of TNF-α, IL-1β, and IL-6 associated with M1 polarization, through an NF-kB-dependent mechanism (54). Expression of these proinflammatory cytokines by bone marrow-derived macrophages was suppressed by RAGE manipulation through genetic deletion or selective inhibition by TTP448 or by NF-kB inhibition by TPCA-1. Altogether, these data identify RAGE as an actionable target to hamper macrophage polarization promoted by cholangiocyte-derived EVs (54). The key role played by macrophages in biliary fibrosis featuring PSC, driven by chemokines and EVs originating from senescent cholangiocytes is illustrated in Figure 1.

### Relationship of Macrophage Activation With Biliary Repair

In PSC, effects of M1-polarized macrophages have also been reported on the level of biliary repair involving hepatic progenitor cells (HPC) (57), whose activation leading to ductular reaction is instrumental in biliary fibrogenesis (4, 16, 58). In cell co-culture studies, bone marrow-derived M1 macrophages promoted a self-renewing phenotype of HPC via activation of the Notch signaling pathway (57). It is important to note that Notch activation in HPC by Jagged1 expressed by myofibroblasts was previously described as a critical pathway promoting HPC differentiation toward the biliary lineage (59). Monocyte/infiltrating macrophage and Notch signaling gene signatures were enriched in PSC liver samples and among core enrichment genes associated with HPC marker Hes Family BHLH Transcription Factor 1 (HES1) (57). Effects of macrophage depletion induced by liposome encapsulated clodronate (a bisphosphonate which inhibits the monocyte-macrophage transdifferentiation) have been extensively investigated in another animal model of PSC, generated by mouse feeding with 3,5-diethoxycarbonyl-1,4-dihydrocollidine (DDC). The DDC-fed mouse is characterized by cholangitis and intense biliary fibrosis associated with macrophage recruitment, activation of portal myofibroblasts, and a prominent ductular reaction (60). In DDC-fed mice, macrophage depletion blunted deposition of laminin, a major component of the basement membrane surrounding the bile ducts. When laminin is defective, reactive ductules were confined within the portal area and did not progress to “atypical” ductules (61), which extend beyond the portal tract boundaries into the parenchyma and represent a feature of a more fibrogenic biliary injury. Moreover, macrophage depletion was accompanied by a significant reduction in the proliferative status of cholangiocytes (61), consistent with previous observations identifying the TNF family member TNF-like weak inducer of apoptosis (TWEAK) as the signal emitted by macrophages that is responsible for expansion of HPC by interacting with its receptor Fn14 and stimulation of ductular reactions by activating NFκB signaling (62, 63). However, macrophage depletion induced by liposomal clodronate involves not only bone marrow-derived macrophages, but also liver-resident macrophages (KCs). A study focusing on the role of F4/80^+^ KCs in the same clodronate-treated DDC model showed that KC depletion was beneficial only in the early phase of biliary injury, while it was detrimental in the regression phase with worsening effects on portal inflammation and fibrosis (64). These data conceivably suggest that KC may play a dual role in sclerosing cholangiopathy in keeping with their marked cell plasticity. Therefore, therapeutic interventions targeting macrophages in sclerosing cholangitis must pay attention to the remarkable heterogeneity of this cell type that cannot be too simplified by the conventional distinction between infiltrating and liver-resident macrophages, and between M1 and M2 phenotypes.

## The Fibrogenic Role of Macrophages in CHF

Fibropolycystic liver diseases, which encompass a number of genetic conditions with biliary dysgenesis such as autosomal recessive polycystic kidney disease (ARPKD), CHF, and Caroli's disease (CD), are caused by mutations in the polycystic kidney and hepatic disease 1 (*PKHD1*), the gene encoding for fibrocystin/polyductin (FPC) (65, 66). FPC is a large protein expressed by ductal epithelial cells, such as cholangiocytes, in several cellular compartments including cilia, centromeres, basal bodies, and tight and adherence junctions (65, 66). Although FPC function is unknown, it is likely involved in cell proliferation, cell-cell and cell-matrix interactions, cell differentiation, secretion, and planar cell polarity (67). A common trait of these diseases is the cyst-like enlargement of the intrahepatic bile duct epithelium accompanied by a progressive deposition of fibrotic tissue in the peribiliary region. In the dysgenetic ducts, cholangiocytes present cilium defects and retain features of immaturity reminiscent of a fetal-like behavior (2–4, 19). Worsening fibrosis leads to portal hypertension and related complications, such as variceal bleeding and ascites, and young patients are generally disposed to develop chronic cholangitis (2, 68, 69). Since effective pharmacological treatments are currently lacking, the only curative approaches are based on surgical interventions such as liver resection (in CD) or liver transplantation when portal hypertension or recurrent acute cholangitis lead to life-threatening complications (70).

### Epithelial Immaturity of Dysgenetic Ducts Is Characterized by an Overactivation of the β-Catenin Signaling to Stimulate Macrophage Recruitment

**Figure 2:**
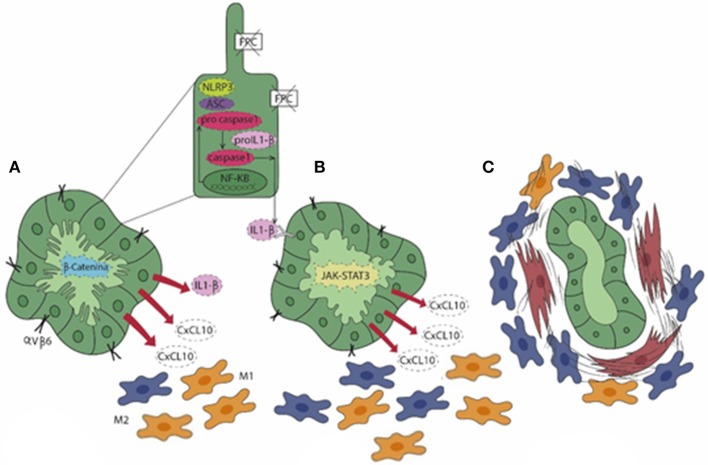
In CHF **(A)**, fibrocystin (FPC)-defective cholangiocytes gain an immature (fetal-like) phenotype characterized by an over-activation of the β-catenin signaling. This enables cholangiocytes to secrete a range of pro-inflammatory chemokines (CXCL1, CXCL10, and CXCL12) and to express integrin αVβ6, the local activator of latent TGFβ, a major driver of liver fibrogenesis. In particular, secretion of CXCL10 is sustained by an autocrine/paracrine loop where the activation of the NLRP3 inflammasome stimulates the secretion of IL-1β via the JAK/STAT3 pathway (pale green cholangiocyte) that, in turn, enhances the production of CXCL10. **(A,B)** The exuberant chemokine secretion by cholangiocytes orchestrates the peribiliary accumulation of macrophages, which are initially molded into the M1 phenotype (yellow) prevailing on the M2 (blue). **(C)** At a later stage, increase in M2 macrophages associates with the recruitment of myofibroblasts (brown), which result in the progressive accumulation of peribiliary fibrotic tissue and in the progression of the disease.

Recent findings suggest that in CHF, biliary fibrosis is the result of a chronic, low-grade inflammatory response sustained by macrophages and originating from FPC-defective cholangiocytes akin to “parainflammation,” a process of adaptation to an unrestrained cell dysfunction. When cell dysfunction is persistent, the inflammatory response, unable to restore the normal tissue homeostasis, becomes pathologic and can ultimately lead to scarring (71). Interestingly, in a murine model of CHF, the *Pkhd1*^*del*4/*del*4^ mouse, macrophages featuring the inflammatory reaction showed a switch of polarization over the disease progression. In fact, macrophages dominating the peribiliary infiltrate in the early stages showed a preponderance of proinflammatory iNOS^+^ M1 macrophages recruited from the circulating precursors that gradually shift to a profibrotic CD206^+^ M2 phenotype as the fibrosis progressed (72). Notably, macrophage infiltration was driven by the hectic secretory functions displayed by FPC-defective cholangiocytes dependent upon an over-activation of the β-catenin signaling (72). This perturbation resulted in an exuberant production of several chemokines, in particular of chemokine (C-X-C motif) ligand 1 (CXCL1), CXCL10, and CXCL12, directing the progressive accumulation of macrophages in the pericystic area. Macrophages in turn released TNF-α when M1-preponderant, coupled with transforming growth factor (TGF) β while enriching in M2, which further stimulated the *de novo* expression of integrin αVβ6 by FPC-defective cholangiocytes. Upregulation of integrin αVβ6, the local activator of latent TGFβ (73), is a crucial mechanism in the peribiliary fibrosis that finally leads to the recruitment of portal myofibroblasts and is actively involved in the generation of the several components of the fibrotic ECM, in particular fibronectin and collagen type I. Thus, in *Pkhd1*^*del*4/*del*4^ mice, biliary fibrosis is initially promoted by macrophages and involvement of portal myofibroblasts occurs only in a later stage, when fibrosis worsens and associates with the development of portal hypertension (72). The translational significance of macrophage targeting in biliary fibrosis even when related to CHF was confirmed by treating *Pkhd1*^*del*4/*del*4^ mice with clodronate *in vivo*. Macrophage depletion was paralleled by a significant reduction in portal fibrosis, which prevented the establishment of portal hypertension (72). Of note, this finding was reproduced in the *cpk/cpk* mice, another model of ARPKD, where macrophage reduction was accompanied by a decreased size of the epithelial cysts not only in the liver, but also in the kidney (74). Besides β-catenin, other signaling anomalies contribute to the pro-inflammatory phenotype activated by FPC deficiency. In fact, the epithelial secretion of CXCL10 is further sustained through an autocrine loop in which the NLRP3 inflammasome complex, once activated, allows the secretion of IL-1β that in turn stimulates CXCL10 secretion through the phosphorylation and activation of the JAK/STAT3 signaling (2, 75). Again, this mechanism is amenable to therapeutic intervention since the *in vivo* treatment of *Pkhd1*^*del*4/*del*4^ mice with AMG-487, an antagonist of CXCR3, the cognate receptor for CXCL10, significantly reduced the extent of M2 infiltrating macrophages together with a significant decrease in the fibrotic and cyst areas (75). Figure 2 summarizes the sequence of events unleashed by FPC inactivation in cholangiocytes, which results in the progressive peribiliary accumulation of macrophages.

### CCL2/MCP-1 Signaling Also Mediates Macrophage Infiltration in Ciliary Dysfunctions

As mentioned, cilium dysfunction represents a defining feature of ductal epithelial cells in these conditions (thereby also termed as “ciliopathies”) (76, 77). A crucial protein essential for cilia formation is the intraflagellar transport 88 (IFT88) (78). Inactivation of the *Ift88* gene in mouse (*IFT88*^*Orpk*^ mouse) leads to liver cyst expansion associated with deposition of fibrosis, which reproduces the liver phenotype of human ARPKD/CHF, similarly to the *Pkhd1*^*del*4/*del*4^ mouse. This model is also characterized by a massive accumulation of Ly-6C^hi^ infiltrating macrophages near the dysgenetic ducts, which preceded the cyst growth and the onset of biliary fibrosis. Macrophage recruitment was mediated by CCL2/MCP-1 expressed by cholangiocytes harboring cilia dysfunction. Phenotyping of peribiliary macrophages showed enhanced gene expression of profibrotic and proinflammatory mediators including IL-6, VEGF-A, TGFβ, and PDGF-B, which in turn directed accumulation and activation of portal fibroblasts in a later stage that promoted fibrosis progression (79). Notably, in this model the genetic manipulation of CCL2 signaling obtained by generating double KO *IFT88*^*Orpk*^/*FBVCCR2*^−/−^ mice caused a significant reduction in Ly-6C^hi^ macrophages and in the pericystic fibrosis, but did not affect liver cyst expansion (79). The relevance of CCL2 signaling was confirmed further in the PCK rat, another well-established rodent model ortholog to the human fibropolycystic liver and kidney diseases. Upon treatment with bindarit, a small molecule that inhibits CCL2 expression and activity, the PCK rat reported a reduction in macrophage accumulation accompanied by an amelioration of renal function, but without significant effects on both liver and kidney cyst growth (80). These data are partially in contrast with results obtained treating inducible *Pkd1*^*fl*/*fl*^*;Pax8-rtTA;TetO-Cre* mice with INCB3344, a specific CCR2 antagonist, which showed a reduction of renal cyst enlargement (81). Collectively, these findings suggest that different mechanisms likely underpin epithelial cyst expansion and biliary fibrosis, and confirm that monocyte-derived macrophages are key drivers of fibrogenesis.

## Conclusions

Mouse models of biliary fibrosis developing in the context of chronic cholangiopathies such as PSC and CHF have revealed that macrophages, in particular the Ly-6C^hi^ population originating from the circulating compartment, hold a crucial role, especially in the initial phase of fibrogenesis. In fact, following an initial bile duct injury, macrophage infiltration of the peribiliary area is an early event preceding the accumulation of the more conventional fibrogenic cell type in the liver, i.e., the portal myofibroblast, ultimately responsible for the deposition of scarring ECM components in the portal area. Interestingly, macrophages are instructed by pro-inflammatory mediators (CCL2, IL-8, CXCL1, CXCL10, CXCL12) or EV-mediated DAMP released by cholangiocytes owing to the gain of a senescent phenotype (as in PSC) or the persistence of an immature, fetal-like ciliary defective phenotype (as in CHF). It would be tempting to speculate on the deep phenotypic similarities that senescence and immaturity, as maturation stages at the opposite ends of the cell life, share at the cholangiocyte level. On the other hand, macrophages populating the peribiliary area show different patterns of polarization over time in the two cholangiopathies. Whilst in PSC, M1 macrophages become preponderant as the disease progresses, in CHF the M1 phenotype dominates the early infiltrate, and is then equated by M2 in a later stage. Nonetheless, macrophages represent ideal targets for drug delivery given their phagocytic functions, and experimental evidence is mounting that their therapeutic targeting provides an attractive strategy to ameliorate biliary fibrosis (21). Of note, this concept is even more relevant in PSC and CHF, which are still orphan diseases and are thus eagerly awaiting novel treatments among cholangiopathies. The relevant therapeutic effects obtained by targeting macrophages in animal models of PSC and CHF are summarized in Table 1. Although these observations may serve as a promising starting point for future therapeutic directions, a number of challenges deserve consideration in future studies. First, it will be important to have a deeper understanding of the variable role of macrophages during disease progression, in particular as it relates to the polarization switch, in order to devise more specific macrophage-targeted interventions. Second, it must be underlined that most data herein discussed have been obtained in rodent models, and thus they need translation to human settings. This is of utmost importance as the heterogeneity of liver macrophages in humans is much less investigated compared to mice. Generation of biliary organoids from patient-derived liver biopsy or bile sample may offer a significant asset to tackle this challenge (82). Finally, additional pathogenetic factors such as microbiota and infections, which are particularly relevant in both PSC and CHF, heavily influence macrophage functions, and thus major efforts will be devoted to address this area.

**Table 1:** Anti-macrophage strategies used for *in vivo* treatments.

**Drug**	**Experimental model**	**Mechanism of action**	**Readouts and therapeutic effects**
Cenicriviroc	C57BL/6. *Mdr2^−/−^* mice	CCR2 antagonist	Reduction in peribiliary infiltration of monocyte-derived macrophages. Improvement of fibrosis and cholestasis.
Clodronate	DDC fed mice, *Pkhd1^*del*4/*del*4^* mice and *cpk/cpk* mice	Inhibitor of monocyte-macrophage transdifferentiation	Reduction of tissue accumulation of M2 macrophages. Significant decrease in fibrosis; reduction in epithelial cyst size in liver and kidney.
AMG-487	*Pkhd1^*del*4/*del*4^* mice	CXCR3 antagonist (CXCL10 receptor)	Reduction of the extent of CD45^+^ leucocytes (mainly M2 macrophages and CD4^+^ T cells). Decrease of the fibrotic area extent and of the cyst size.
Genetic inhibition of CCL2 signaling	Double KO *IFT88Orpk/FBVCCR2^−/−^* mice	CCL2 signaling inhibitor	Significant reduction of Ly6c^hi^ macrophages. Improvement of fibrosis.
Bindarit	PCK rat	Inhibitor of CCL2 expression/activity	Significant reduction of macrophage accumulation. Amelioration of renal function.
INCB3344	Inducible *Pkd1fl/fl;* *Pax8-rtTA; TetO-Cre* mice	CCR2 antagonist	Reduction of renal cyst enlargement.

## Author Contributions

MC designed the study and wrote the first draft. NG wrote the first draft. GG and MS critically reviewed the draft and finalized the manuscript. LF designed the study, critically reviewed the draft and finalized the manuscript.

## Conflict of Interest

MS is member of the advisory board of Esiai/Merck, Bayer and Engitix. The remaining authors declare that the research was conducted in the absence of any commercial or financial relationships that could be construed as a potential conflict of interest.
